# Challenges in Diagnosing Primary Intracranial Ewing Sarcoma/Peripheral Primitive Neuroectodermal Tumor: A Case Report

**DOI:** 10.2174/0115734056334823241216180031

**Published:** 2025-01-02

**Authors:** Shigang Luo, Feifei Wang, Huan Haung, GuangCai Tang

**Affiliations:** 1 Department of Radiology, The First People's Hospital of Guang Yuan, Guang Yuan, China; 2 Department of Radiology, Affiliated Hospital of Southwest Medical University, Luzhou, China

**Keywords:** Ewing sarcoma, Peripheral primitive neuroectodermal tumors, Diagnosis, Treatment, headache

## Abstract

**Background:**

Primary intracranial Ewing Sarcoma/peripheral Primitive Neuroectodermal Tumor (EWS/pPNET) is exceedingly rare and easy to misdiagnose.

**Case Presentation:**

We present a case involving a 23-year-old male who presented with headaches and vomiting. The preoperative brain imaging revealed an irregular mass in the left parietal lobe, initially misdiagnosed as meningioma. However, the surgical specimen was ultimately diagnosed as primary intracranial EWS/pPNET. The patient underwent a total tumor resection, followed by adjuvant chemotherapy and radiotherapy. No recurrence or distant metastasis was observed 18 months after the surgery.

**Conclusion:**

When the imaging features of young patients’ lesions are solid, aggressive, and unevenly enhanced masses, physicians should be aware of the possibility of primary intracranial EWS/pPNET, and if possible, Gross Total Resection (GTR) and intensive chemotherapy and radiotherapy are recommended.

## 1 INTRODUCTION

Primary intracranial EWS/pPNET is exceedingly rare, appearing as undifferentiated, small, round, blue-cell neoplasms, originating from long bones and soft tissue. It is the second most common primary malignant bone tumor in children and adolescents [1-3]. The most common chromosome translocation in EWS/pPNET is chromosome t(11,22) (q24;q12) translocation, which accounts for approximately 90% of EWS/pPNET cases [4]. The primary intracranial location is extremely rare, with only around 100 cases reported. Primary intracranial EWS/pPNET differs from central Primitive Neuroectodermal Tumors (cPNET) in terms of potential genetics, treatment, and prognosis [5, 6]. Given the rarity of primary intracranial EWS/pPNET, particularly regarding prognosis and treatment, we, herein, report our experience.

## 2 CASE PRESENTATION

**Fig. (1):**
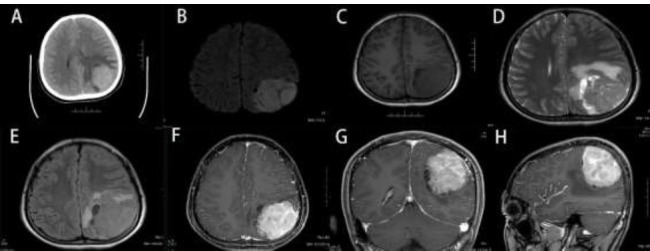
Preoperative CT and MRI demonstrate a large contrast-enhancing tumor in the left parietal lobe. (**A**) Axial CT. (**B**) Diffusion-weighted imaging axial MRI. (**C**) T1-weighted axial MRl. (**D**) T2-weighted non-contrast axial MRl. (**E**) Fluid Attenuated Inversion Recovery (FLAIR)-weighted axial MRI. (**F**) T1-weighted contrast-enhanced axial MRI. (**G**) T1-weighted contrast-enhanced coronary MRI. (**H**) T1-weighted contrast-enhanced sagittal MRI.

A 23-year-old male presented to the hospital with a 1-week history of headache and vomiting. The patient exhibited neither neurological deficits nor signs or symptoms of systemic illness. His medical history and family history were normal. The physical examination and laboratory examinations were normal. CT revealed an irregular mass with a slightly higher density with clear borders in the left parietal lobe with surrounding edema (Fig. **1**). MRI discovered a large space-occupying lesion of the left parietal lobe measuring 5.8×4.5×6.1cm. The lesion displayed isointense-to-hypointense signals on a T1WI scan, and T2WI exhibited hypointense-to-hyperintense signals (Fig. ** 1**). The tumor mass appeared to have invaded and disrupted the dura mater, and after contrast, the lesion showed a heterogeneous enhancement (Fig. ** 1**). The preoperative clinical diagnosis was a meningioma, and the patient underwent surgery to remove the lesion. The tumor specimen was grayish-white, firm, rich in blood supply, had a specific boundary with the surrounding brain tissue, and had invaded the adjacent dura mater. The lesion was completely resected with no residue.

**Fig. (2):**
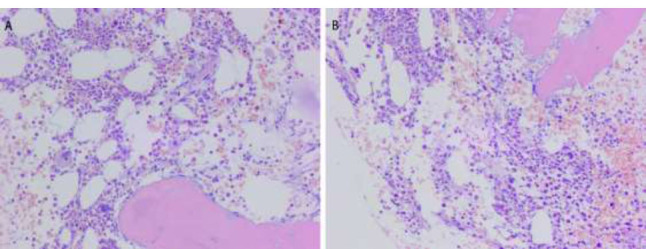
(**A**, **B**) H&E staining showing a small blue cell tumor.

**Fig. (3):**
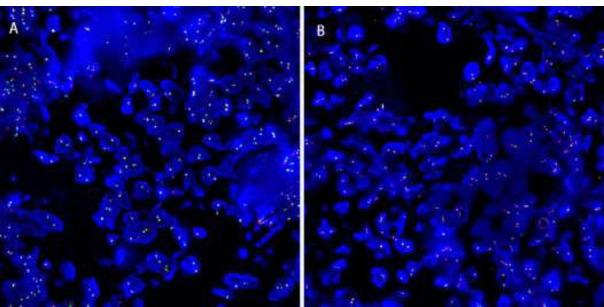
(**A**, **B**) FISH assay showing EWSR1 rearrangement.

Histological Analysis: The tumor was composed of small round cells with hyperchromatic nuclei, scant cytoplasm, and granular chromatin (Fig. ** 2**). Immunohistochemical exami- nation revealed that the neoplastic cells were positive for CD99, FLI1, INI, and Syn, and negative for S-100, myoD1, CGa, GFAP, LCA, TdT, CK, EMA, and TLE (Fig. ** 2**). Moreover, the genetic analysis was positive for the EWSR1 rearrangement diagnostic of EWS and showed > 20% of split signals confirming the diagnosis of primary intracranial EWS/pPNET (Fig. ** 3**).

**Fig. (4):**
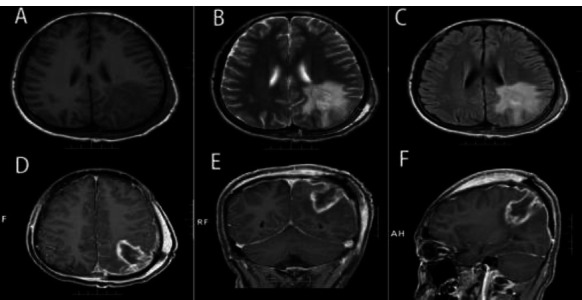
(**A**-**F**) The patient underwent an MRI examination one week after the surgery, and no tumor residue was found.

**Fig. (5):**
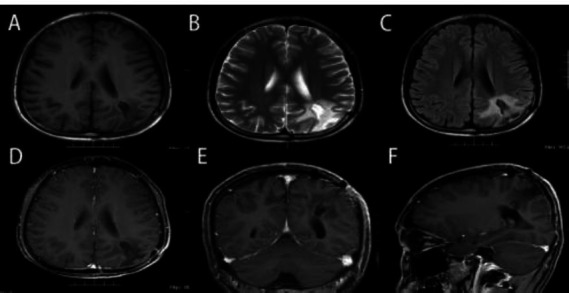
(**A**-**F**) After surgery, the patient underwent radiation therapy and chemotherapy. After 18 months, an MRI examination was performed, and no residual tumor or recurrence was found.

The postoperative period was uneventful, and a CT of the thorax and abdomen was performed, as well as bone scans; all results were negative. One week after the operation, the brain MRI indicated that the lesion had been entirely removed, with no residual or recurrent tumor evidence (Fig. **4**). The patient’s parents opted for adjuvant therapy, including chemotherapy and focal radiation. Chemotherapy drugs included vincristine, cyclophosphamide, and doxorubicin, alternating with ifosfamide and etoposide, followed by focal radiation with a total dose of 50 Gy. During the final follow-up, 18 months after diagnosis, the patient’s imaging features indicated no evidence of recurrence or metastasis (Fig. **5**).

## 3 DISCUSSION

James Ewing was the first to report Ewing's Sarcoma (EWS) and theorized that EWS originated from the vascular endothelium [7]. Primary intracranial EWS/pPNET is exceedingly rare, and appears as undifferentiated, small, round, blue-cell neoplasms, originating from long bones and soft tissue. In 2002, the World Health Organization classified EWS and peripheral Primitive Neuroectodermal Tumor (pPNET) as a single pathological entity [8]. EWS/pPNET is the second most common primary bone tumor after osteosarcoma in adolescents and children, which usually occurs in the long bones of limbs and pelvis and most often appears in the second decade of life. There are no significant gender differences [9, 10]. Primary intracranial locations are extremely rare, with only approximately 100 cases of primary intracranial EWS/pPNET reported in English studies, with a relatively high number of male patients [11, 12]. The clinical presentation of primary intracranial EWS/pPNET is variable, usually determined by the tumor’s site, location, and size. In the reported cases, headache and vomiting are the most common clinical symptoms related to increased Intracranial Pressure (ICP), followed by cranial nerve damage, seizures, and a cranial mass [13, 14]. In this case, the main symptoms were headache and vomiting, which are common signs of raised ICP.

In this case, the imaging revealed an irregular mass in the left parietal lobe with heterogeneous enhancement, which could easily be mistaken for more common intracranial tumors, such as meningioma. Previous studies have demonstrated that the tumor commonly exhibits iso-to-hypointense signals on T1WI scans and iso-to-hyperintense signals on T2WI scans. In terms of enhancement, it is typically moderate, heterogeneous, or intense, with heterogeneous enhancement being the most common [13]. However, due to the non-specific and variable radiological characteristics, it is difficult to make any confident radiological diagnosis; it is easily misdiagnosed as rhabdomyosarcoma hemangiopericytoma or metastatic tumors, especially malignant meningioma.

Despite the imaging findings, the final diagnosis depended on immunohistochemistry and genetic analysis, particularly CD99 positivity and EWSR1 rearrangement [15]. Previous studies have reported that the MIC2 gene product (CD99) immune expression is important for EWS/PNET diagnosis [16]. The sensitivity of CD99 is 93%, and the specificity is 80% [17]. A central Primitive Neuroectodermal Tumor (cPNET) that is negative for the MIC2 gene and CD99 can be used in the differential diagnosis [18]. However, CD99 can be found in other small blue round cell tumors, including lymphoma, neuroblastoma, and rhabdomyosarcoma [18, 19]. Thus, at present, the final diagnosis must be confirmed by the translocation of the EWS gene. The most common translocation is t(11, 22) (EWS-FLI-1), which accounts for >90% of EWS/pPNET. The second is ESW/ERG t(21, 22) (EWS-ERG), with less common translocations, including EWS-ETV1, EWS-E1AF, and EWS-FEV, with an incidence of each case being less than 1% [20]. Consistent with previous studies, our case was confirmed as EWS/pPNET through immunohistochemical marker CD99 and genetic testing for EWSR1 rearrangement.

The current treatment recommendation from the National Comprehensive Cancer Network (NCCN) is local treatment (surgery and/or radiotherapy) plus chemotherapy [20]. A Gross Total Resection (GTR) can improve patient quality of life, improve long-term survival, and reduce local recurrence. The prognosis of patients who did not receive GTR was worse than those who received GTR [11, 13]. In our case, the patient underwent GTR, followed by adjuvant chemotherapy and radiotherapy, and thus far, no indication of recurrence or metastasis has been reported. Chen *et al*. [14] reported that patients who received partial resection had a shorter median survival time compared to those who underwent GTR. Chemotherapy is one of the most important treatments for primary intracranial EWS/pPNET. A previous study showed that adjuvant chemotherapy improved the 5-year survival rate from 5-10% to over 65% [21-23]. Current systemic chemotherapy drugs include vincristine-doxorubicin-cyclophosphamide and dactinomycin alternating with ifosfamide-etoposide [13]. Local radiotherapy is generally used for tumors that cannot be removed by surgery or residual tumors. Chen *et al*. [14] reported that adjuvant radiotherapy could improve the patients’ 1-year and 2-year survival rates (from 60.0% to 88.9% and 0 to 66.7%, respectively). We observed that patients undergoing surgery, radiotherapy, and chemotherapy tended to best prognosis. Michael *et al*. [24] reported that a young female patient who received GTR, systemic chemotherapy, and focal radiation had a survival time of 10.5 years.

The risk factors associated with worse prognosis in primary intracranial EWS/pPNET include tumor location, surgical margins, radiotherapy, chemotherapy, and with or without metastasis. Distant metastasis is a poor prognostic factor, with extrapulmonary metastases having the worst prognosis [25]. However, age, gender, and the occurrence of symptoms do not influence the prognosis [11, 13-15]. Previous studies suggest that patients who undergo surgery, radiotherapy, and chemotherapy may achieve better long-term disease-free survival. Our case supported this as the patient remained disease-free during follow-up.

Due to the rarity of primary intracranial EWS/pPNET, there is limited understanding of optimal treatment and long-term outcomes. This case report was limited by its single case and relatively short follow-up period. Future research should focus on long-term studies with larger cohorts to further clarify the prognosis and best treatment strategies for this rare tumor.

## CONCLUSION

Primary intracranial EWS/pPNET is rare and often misdiagnosed. Genetic evaluation is the gold standard for primary intracranial EWS/pPNET diagnosis. When the imaging features of young patients’ lesions are solid, aggressive, and unevenly enhanced masses, physicians should be aware of this rare tumor. If possible, GTR combined with intensive chemotherapy and radiotherapy is recommended.

## Data Availability

The data and supportive information are available within the article.

## LIST OF ABBREVIATIONS

EWS: Ewing sarcoma
pPNET: Peripheral primitive neuroectodermal tumor
cPNET: Central primitive neuroectodermal tumor
GTR: Gross total resection
CT: Computed tomography
MRI: Magnetic resonance imaging
CD99: Anti-CD99 antibody
EWSR1: Ewing sarcoma breakpoint region 1 gene.
Gy (Gray): Absorbed dose
H&E: Hematoxylin-eosin staining
ICP: Intracranial pressure
T1WI: T1 weighted imaging
T2WI: T2 weighted imaging
NCCN: The National Comprehensive Cancer Network

## STANDARDS OF REPORTING

CARE guidelines were followed.
